# Supplementary material for: Network approach reveals preferential T-cell and macrophage association with α-linked β-cells in early stage of insulitis in NOD mice
Source: Front Netw Physiol. 2024 Jun 24;4:1393397. doi: 10.3389/fnetp.2024.1393397 (PMC11228247; doi:10.3389/fnetp.2024.1393397)
Supplement: Supplementary file 1 [file DataSheet1.docx]

Supplementary Material

Network approach reveals preferential T-cell and macrophage association with α-linked β-cells in early stage of insulitis in NOD mice.

Nirmala V. Balasenthilkumaran, Jennifer C. Whitesell, Rachel S. Friedman, Laura Pyle, Vira Kravets^*^

*** Correspondence:** Vira Kravets: [vkravets@ucsd.edu](mailto:vkravets@ucsd.edu)

# Supplementary Figures

**

**

**Supplementary Figure S1.** Mander’s colocalization coefficients (M1 and M2) of the immune cell markers (CD3, CD11c, and F4/80) utilized in our study (n = 86 islets from 8 mice).





**Supplementary Figure S2.** Information on age, blood glucose recordings, number of islets analyzed, and the range of insulitis degrees of all the mice utilized in our study.





**Supplementary Figure S3.** **(A)** Comparison of K_avg_ of different α-cell networks (created using different thresholds (thr)) using network analysis (n = 134 islets from 11 mice). **(B)** Comparison of K_avg_ of different immune cell networks (created using different thresholds) using network analysis (n = 134 islets from 11 mice). RM one-way ANOVA with Geisser-Greenhouse correction and Tukey's multiple comparisons test was used for statistical analysis in A and B. **(C)** CD11c signal of the islet shown in Figure 1B before pre-processing. **(D)** CD11c signal of the islet shown in Figure 1B after pre-processing and bleed-through correction. **(E)** Relationship between the node degrees of different β – immune cell networks obtained using network analysis (different thresholds) (n = 134 islets from 11 mice) (Simple linear regression was used for statistical analysis).





**Supplementary Figure S4.** **(A)** Variation of T-cell density of the islets with insulitis pseudotime (T/β cell ratios sorted in ascending order) (n = 134 islets from 11 mice) (Non-linear regression curve fit was used for statistical analysis). **(B)** T-cell density curve in A fit using a log scale (n = 134 islets from 11 mice). **(C)** Comparison of insulitis degrees of islets classified as early- (n = 44 islets), intermediate- (n = 46 islets), and late- (n = 44 islets) stage insulitis. **(D)** Insulitis pseudotime in Figure 2B fit using a log scale (n = 134 islets from 11 mice). **(E)** Relationship between the number of β-cells and the number of T-cells in each islet (n = 134 islets from 11 mice). **(F)** Progression of insulitis of different islets in a single mouse sorted using T/β cell ratios (pseudotime) (E - early-stage insulitis, I - intermediate stage insulitis, L – late-stage insulitis). Simple linear regression was used for statistical analysis in B, D, and E.





**Supplementary Figure S5**. **(A)** Schematic representation of the algorithm used to divide an islet into two halves. **(B)** Relationship between the proportion of α-cells and the proportion of immune cells at a distance of at a distance of 20 μm from the islet rim in half islets (n = 134 islets from 11 mice) (horizontal division). **(C)** Relationship between the proportion of β-cells and the proportion of immune cells at a distance of 20 μm from the islet rim in half islets (n = 134 islets from 11 mice) (vertical division). **(D)** Relationship between the proportion of β-cells and the proportion of immune cells at a distance of at a distance of 20 μm from the islet rim in half islets (n = 134 islets from 11 mice) (horizontal division). Simple linear regression was used for statistical analysis in B, C, and D.





**Supplementary Figure S6**. α-cell and T-cell positions of a representative early-stage islet, and 10 seeds of the representative islet randomized using the proposed randomization algorithm.





**Supplementary Figure S7**. **(A)** Experimental and random α-cell – T-cell networks of a representative early-stage islet (positions of α-cells were randomized). **(B)** Comparison of K_avg_ of experimental and random α-cell – T-cell networks (nwks) in early- (n = 44 islets), intermediate- (interm) (n = 46 islets), and late- (n = 44 islets) stages using network analysis. **(C)** Experimental and random α-cell – macrophage (Mϕ) networks of a representative early-stage islet (positions of α-cells were randomized). **(D)** Comparison of K_avg_ of experimental and random α-cell – macrophage networks in early- (n = 44 islets), intermediate- (n = 46 islets), and late- (n = 44 islets) stages using network analysis. **(E)** Experimental and random α-cell – myeloid (mye) cell networks of a representative early-stage islet (positions of α-cells were randomized). **(F)** Comparison of K_avg_ of experimental and random α-cell – myeloid cell networks in early- (n = 44 islets), intermediate- (n = 46 islets), and late- (n = 44 islets) stages using network analysis. Paired parametric t- tests were used for statistical analysis in B, D, and F. See Materials and Methods for detailed description on of randomization and network analysis.





**Supplementary Figure S8**. **(A)** Comparison of K_avg_ of experimental and random α – T cell networks in early- (n = 44 islets), intermediate- (n = 46 islets), and late- (n = 44 islets) stages using network analysis (different thresholds (thr)). **(B)** Comparison of K_avg_ of experimental and random α-cell – macrophage (Mφ) networks in early- (n = 44 islets), intermediate- (n = 46 islets), and late- (n = 44 islets) stages using network analysis (different thresholds). **(C)** Comparison of K_avg_ of experimental and random α – myeloid (mye) cell networks in early- (n = 44 islets), intermediate- (n = 46 islets), and late- (n = 44 islets) stages using network analysis (different thresholds). Paired parametric t-tests were used for statistical analysis in A-C.





**Supplementary Figure S9**. α-, β- and T-cell positions of 10 randomly picked early-stage islets.





**Supplementary Figure S10**. **(A)** Progression of insulitis in different islets, sorted using T/β cell ratios (pseudotime) (n = 134 islets from 11 mice). **(B)** Comparison of K_avg_ of small-sized experimental and random α – T cell, and experimental and random α-cell – macrophage (Mφ) networks in early- (n = 16 islets), intermediate- (n = 13 islets), and late- (19 islets) stages using network analysis. **(C)** Comparison of K_avg_ of medium-sized experimental and random α – T cell, and experimental and random α-cell – macrophage (Mφ) networks in early- (n = 17 islets), intermediate- (n = 13 islets), and late- (n = 16 islets) stages using network analysis. **(D)** Comparison of K_avg_ of large-sized experimental and random α – T cell, and experimental and random α-cell – macrophage (Mφ) networks in early- (n = 11 islets), intermediate- (n = 20 islets), and late- (n = 9 islets) stages using network analysis. Paired parametric t-tests were used for statistical analysis in B-D.





**Supplementary Figure S11**. **(A)** Comparison of K_avg_ of α-linked β-cell – and non-α-linked β-cell – T cell networks in early-, intermediate-, and late- stages using network analysis (different thresholds (thr)). **(B)** Comparison of K_avg_ of α-linked β-cell – and non-α-linked β-cell – macrophage (Mφ) networks in early-, intermediate-, and late- stages using network analysis (different thresholds). **(C)** Comparison of K_avg_ of α-linked β-cell – and non-α-linked β-cell – myeloid (mye) cell networks in early-, intermediate-, and late- stages using network analysis (different thresholds). Wilcoxon matched-pairs signed rank test was used for statistical analysis in A-C.





**Supplementary Figure S12**: **(A)** Progression of insulitis in different islets, sorted using T/β cell ratios (pseudotime) (n = 134 islets from 11 mice). **(B)** Comparison of K_avg_ of small-sized α-linked β-cell – and non-α-linked β-cell – T cell, and α-linked β-cell – and non-α-linked β-cell – macrophage (Mφ) networks in early- (n = 14 islets), intermediate- (n = 12 islets), and late- (n = 12 islets) stages using network analysis. **(C)** Comparison of K_avg_ of medium-sized α-linked β-cell – and non-α-linked β-cell – T cell, and α-linked β-cell – and non-α-linked β-cell – macrophage (Mφ) networks in early- (n = 17 islets), intermediate- (n = 13 islets), and late- (n = 14 islets) stages using network analysis. **(D)** Comparison of K_avg_ of large-sized α-linked β-cell – and non-α-linked β-cell – T cell, and α-linked β-cell – and non-α-linked β-cell – macrophage (Mφ) networks in early- (n = 11 islets), intermediate- (n = 20 islets), and late- (n = 9 islets) stages using network analysis. Wilcoxon matched-pairs signed rank test was used for statistical analysis in B-D.
